# Synthesis and Characterization of Novel Hydrazone Complexes: Exploring DNA/BSA Binding and Antimicrobial Potential

**DOI:** 10.1021/acsomega.5c00069

**Published:** 2025-02-13

**Authors:** Jeniffer Meyer Moreira, Sara dos Santos Félix Vieira, Gabriel de Deus Correia, Leandro Nascimento de Almeida, Simone Finoto, Cândida Alíssia Brandl, Aujenus Albert Msumange, Fernanda Galvão, Kelly Mari Pires de Oliveira, Guilherme Caneppele Paveglio, Monize Martins da Silva, Bárbara Tirloni, Cláudio Teodoro de Carvalho, Daiane Roman

**Affiliations:** † Quality Control and Thermal Analysis Laboratory, Federal University of Grande Dourados, Dourados, State of Mato Grosso do Sul 79804-970, Brazil; ‡ Molecular Synthesis and Modification Laboratory, Federal University of Grande Dourados, Dourados, State of Mato Grosso do Sul 79804-970, Brazil; § Department of Chemistry, Federal University of Santa Maria, Santa Maria, Rio Grande do Sul 97105-900, Brazil; ∥ Federal University of Grande Dourados, Dourados, Mato Grosso do Sul 79804-970, Brazil; ⊥ Hydraulics and Environmental Sanitation Laboratory, State University of Mato Grosso do Sul, Dourados, Mato Grosso do Sul 79804-970, Brazil; # State University of Amapá, Macapa, Amapá 68900-070, Brazil

## Abstract

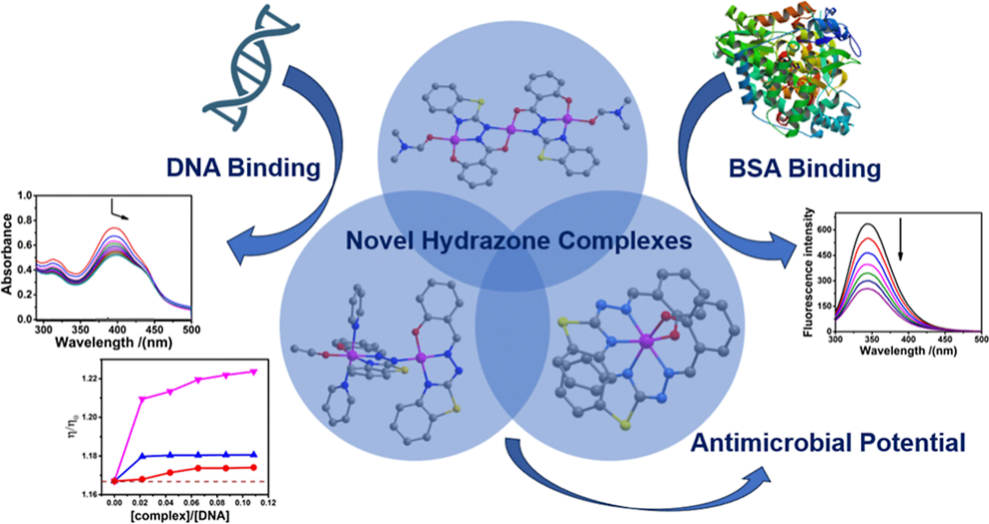

Research involving coordination chemistry with Schiff base hydrazones finds applications in various areas, particularly in bioinorganic chemistry and biomedicine. This work aims to contribute to this field by employing the ligand (E)-2-((2-(benzothiazol-2-yl)hydrazone)methyl)phenol (H_2_L), synthesized via a condensation reaction with salicylic aldehyde. The ligand was isolated, characterized, and subsequently complexed with nickel(II) chloride and copper(II) nitrate, yielding three new crystalline complexes: [Ni(HL)_2_] (**1**), [Ni_2_(L)_2_(Py)_2_(EtOH)]·DMF·0.5H_2_O (**2**), and [Cu_3_(L^#^)_2_(DMF)_2_] (**3**) (where Py = pyridine). The metal complexes were structurally characterized using IR, UV–vis, TGA-DSC, and SCXRD techniques. These analyses confirmed the coordination of the ligand to the metal center via nitrogen and oxygen donor atoms, establishing the formation of mono-, bi-, and trinuclear complexes, respectively. DNA interaction studies were performed through spectroscopic titration and viscosity measurements, indicating that the complexes interact via an intercalative mode, with the interaction order being **3** > **2**> **1**. Partition coefficient analysis revealed that complexes **1** and **3** have a greater tendency to partition into the organic phase, suggesting their potential to cross lipid membranes, while complex **2** and the ligand are more hydrophilic. Fluorescence-based BSA binding studies demonstrated interactions between the complexes and the biomolecule, following the same order as observed in the DNA interaction. Biological tests showed that the ligand lacked antimicrobial and antiyeast activity, while the metal complexes are biologically active. Notably, the copper complex displayed the strongest antibacterial effect, likely due to copper’s essential biological role.

## 1 Introduction

Benzothiazole is a heterocyclic compound distinguished by its bicyclic ring structure, which consists of a benzene ring fused with 4,5 positions of thiazole ring.^1^ The initial biological studies conducted in the 1950s on derivatives of this target molecule explored its potential application as a muscle relaxant.^2^ Since then, this class of heterocyclic compounds has attracted considerable attention in research due to a wide range of relevant biological applications such as anticancer, antiviral, antileishmanial, and antimicrobial agent.^1,3−6^

Schiff bases, or hydrazones (R_2_C = NNR_2_), represent another important class of biologically active organic compounds that have been extensively studied in medicinal chemistry.^7^ The synthesis of hydrazones employs simple, easy-to-handle reagents and involves the condensation of hydrazine with an aldehyde or ketone.^8^ In this sense, hydrazones containing the benzothiazole group can be accessed through the condensation between 2-hydrazinobenzothiazole and aromatic aldehydes. The association of the benzothiazole and hydrazone moiety to form a unique molecular system (Bzt–NH–N = CH–Aryl; Bzt: benzothiazolyl) can lead to a structure with important biological properties.^9^ For example, hydrazones synthesized from 2-hydrazinobenzothiazoles and aromatic aldehydes bearing −OH substituents have exhibited promising anticancer action and have been recognized as potential lead compounds for the development of anticancer drugs.^10^ In another study, the pharmacogenomic approach identified a hydrazone containing the benzothiazole moiety as a candidate for new therapies against multidrug resistant (MDR) cancer. Additionally, structure–activity relationships analysis revealed that donor chelators, such as benzothiazoles, exhibited superior antiproliferative activity compared to other derivatives.^11^

Despite the optimal biological answer of hydrazones containing the benzothiazole core, the complexes synthesized also have several biological applications such as antifungal, antibacterial, antituberculosis, antitrypanosomal, anticancer and antioxidant.^12−16^ Mahale and co-workers synthesized hydrazones from 2-hydrazinobenzothiazole and salicylaldehyde, which were coordinated with molybdenum salts. All complexes showed superior results against the fungi tested and equal or better for the bacteria tested than the free ligand.^13^ Similarly, Wardell and co-workers coordinated the ligand 2-(benzothiazol-2-yl-hydrazonomethyl)phenol with copper salts and these complexes demonstrated more potent antituberculosis activity than the standard drug ethambutol in *in vitro* studies.^14^

This biological potential highlights the importance of hydrazone complexes and supports the need for further research to identify more potent derivatives. As part of our ongoing research into complexes incorporating hydrazones as ligands and their applications in biological studies, we report the synthesis and characterization of three new complexes from the coordination of ligand 2-(benzothiazol-2-yl-hydrazonomethyl)phenol with copper or nickel salts. The characterization of these complexes was performed using thermal analysis (TGA), spectral methods (IR and NMR), and X-ray diffraction for structural determination. Furthermore, this work evaluated the antimicrobial potential of the complexes and also their interaction with DNA/BSA. Studying DNA interactions is crucial, as it provides an understanding of the potential for these complexes to interfere with cellular replication processes, a key property in anticancer agents. Similarly, analyzing the binding affinity with BSA is essential to understanding the transport and bioavailability of these compounds within the bloodstream, as BSA is the primary carrier protein for many drugs in the circulatory system.^17^

## 2 Experimental Section

### 2.1 Instrumental Analysis

The TGA-DSC was performed using a Netzsch STA449 F3 Jupiter thermogravimetric analyzer with sample masses of approximately 4.0 mg and α-alumina crucibles. The purge gas (air) flow rate was set to 50 mL min^–1^, and the heating rate was 10 °C min^–1^ furnace a temperature range of 30–1000 °C. The IR spectra of all materials were analyzed by using a JASCO FT/IR-4100. The samples were prepared in predried KBr pellets and recorded in 4000–400 cm^–1^. UV–vis spectroscopy analysis was conducted using a Varian Cary 50 UV–vis spectrophotometer, with quartz cuvettes having an optical path length of 1.0 cm. All samples were dissolved in DMF. The melting points (mp) of all synthesized compounds were measured in PFM-II 9528/1312 equipment. The ^1^H and ^13^C NMR spectra were recorded on the Bruker Avance II 400 Spectrometer (400 MHz for ^1^H and 100 MHz for ^13^C). The ^1^H and ^13^C data were obtained using DMSO-*d*_6_ as solvent and tetramethylsilane (TMS) as an internal reference.

Data collection for the complexes was performed using either a Bruker D8 Venture diffractometer with Mo Kα radiation (0.71073 Å) or a Bruker D8 Quest diffractometer with Cu Kα radiation (1.54178 Å). The structures were solved using the Intrinsic Phasing method and refined with the ShelXle program.^18^ Refinements were performed using the ShelX program package through full-matrix least-squares analysis of the structural factors (F^2^), applying anisotropic thermal displacement parameters to all nonhydrogen atoms.^19^ Hydrogen atoms were included in the refinement at calculated positions, based on the overall geometry of the molecular fragments, and modeled as groups attached to the corresponding nonhydrogen atoms. Graphical representations of the crystal structures were created using the Diamond program.^20^ Crystal data and further details regarding the data collection and refinement for **1**–**3** are shown in Table S1. CCDC 2405096, 2404847, and 2404848 contain the supplementary crystallographic data for **1**–**3**. These data can be obtained free of charge at http://www.ccdc.cam.ac.uk; from the Cambridge Crystallographic Data Centre at 12 Union Road, Cambridge CB2 1EZ, UK; via fax: (+44) 1223–336–033; or via e-mail: deposit@ccdc.cam.ac.uk.

### 2.2 Synthesis of Precursor, Ligand and Complexes

**Scheme 1:**
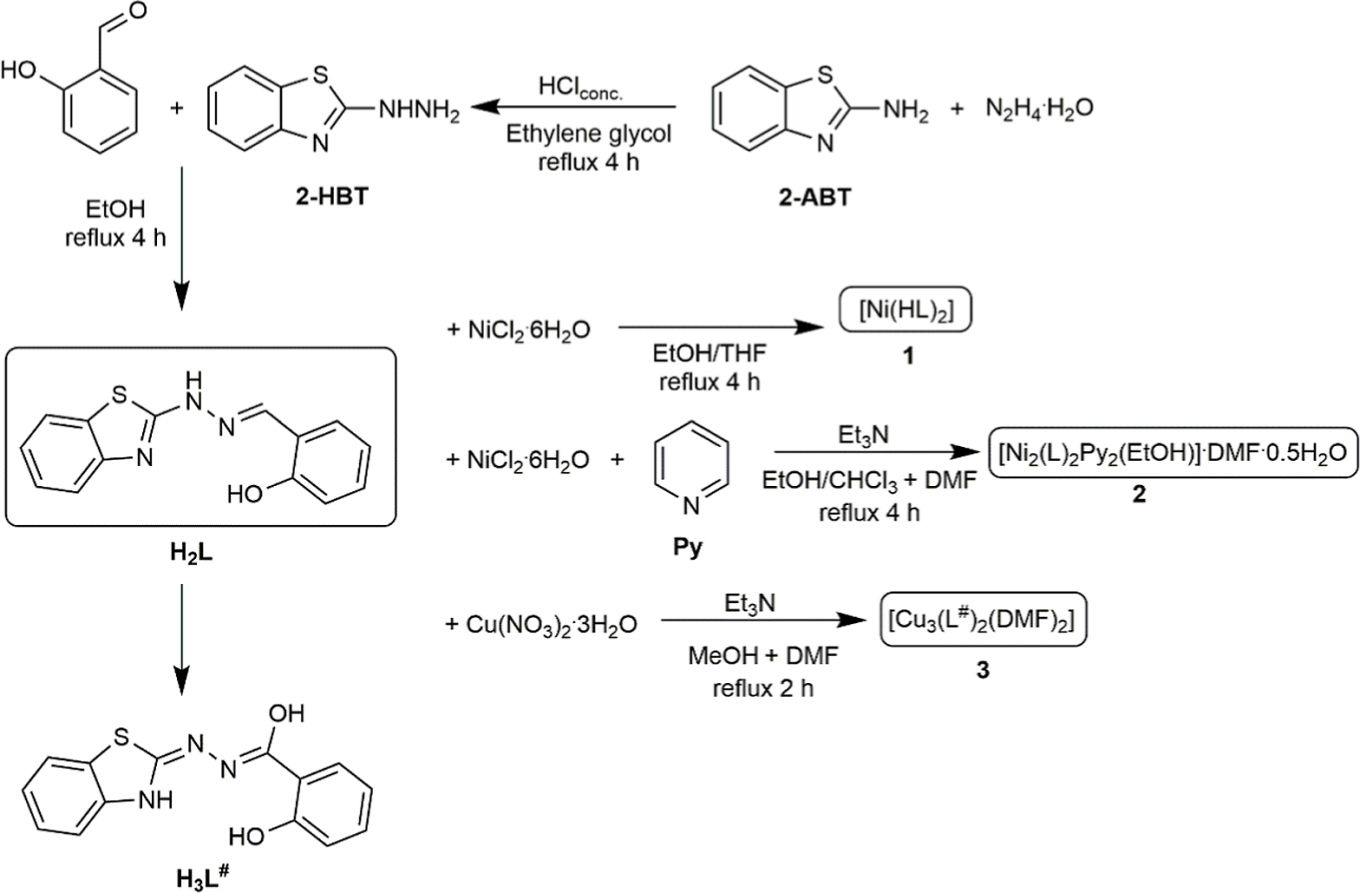
Simplified Scheme of 2-HBT, Ligand and Complexes Synthesis

The solvents and reagents used in this work were purchased from commercial suppliers (Vetec/Sigma-Aldrich) and were used without further purification. The 2-hydrazinobenzothiazole (2-HBT), the ligand (H_2_L), and their corresponding complexes were synthesized by adapting procedures from the literature,^13,21−23^ as depicted in Scheme 1. All experiments were conducted under reflux with stirring.

#### 2.2.1 2-Hydrazinobenzothiazole (2-HBT)

The 2-HBT was synthesized by first mixing 2.0 mL (40 mmol) of hydrazine monohydrate with 2 mL of concentrated HCl at a temperature of 5–10 °C, resulting in the formation of a white solid. After stirring for 15 min, 1500 mg (10 mmol) of 2-aminobenzothiazole (2-ABT) was dissolved in 8 mL of ethylene glycol and added to the mixture. The solution was then refluxed (140 °C) for 4 h, yielding a clear solution. Upon cooling and the addition of crushed ice, a gray solid precipitated, which was subsequently filtered, washed with distilled water, and dried. Molecular formula C_7_H_7_N_3_S (165.04 g mol^–1^). Yield: 93%; mp: 198 °C, according to the literature.^12^ IR (KBr pellet, cm^–1^): 3318 ν(N–H); 3200 and 3129 ν(N–H); 3060 ν(C–H)_ar_; 1650 ν(C=N); 985 ν(N–N). UV–vis (DMF; λ_max_ /nm): 300. ^1^H NMR (400 MHz, DMSO-*d*_6_): δ (ppm) 9.00 (s, 1H); 7.67 (dd, *J* = 0.8, 7.8 Hz, 1H); 7.33 (dd, *J* = 0.5, 8.0 Hz, 1H); 7.20 (td, *J* = 1.3, 7.7 Hz, 1H); 6.98 (td, *J* = 1.2, 7.7 Hz, 1H); 5.00 (s, 2H). ^13^C NMR (100 MHz, DMSO-*d*_6_): δ (ppm) 173.8; 153.4; 130.4; 125.3; 120.9; 120.3; 117.9.

#### 2.2.2 (E)-2-((2-(benzo[*d*]thiazol-2-yl)hydrazone)methyl)phenol (H_2_L)

The H_2_L ligand was synthesized via a condensation reaction, following a modified version of the procedure described in the literature.^13^ In a 50 mL round-bottom flask, 220.0 mg (1.33 mmol) of 2-hydrazinobenzothiazole and 162.0 mg (1.33 mmol) of salicylic aldehyde were dissolved in 12 mL of ethanol under stirring and heating to 70 °C. The reaction mixture was then refluxed for 4 h. After cooling, the resulting solid was collected by simple filtration. Molecular formula C_14_H_11_N_3_OS (269.32 g mol^–1^). Yield: 87%; mp: 259 °C, according to the literature.^12^ IR (KBr pellet, cm^–1^): 3213 ν(N–H); 3043 ν(C–H)_ar_; 2971 ν(O–H); 1623 ν(C=N); 1573 ν(C=N)_ring_; 1276 ν(C–O). UV–vis (DMF; λ_max_ /nm): 358. ^1^H NMR (400 MHz, DMSO-*d*_6_): δ (ppm) 12.13 (s br, 1H); 10.47 (s br, 1H); 8.46 (s, 1H); 7.73 (d, *J* = 7.7 Hz, 1H); 7.61 (dd, *J* = 1.4, 7.7 Hz, 1H); 7.36 (d, *J* = 7.9 Hz, 1H); 7.24–7.31 (m, 2H); 7.09 (td, *J* = 1.1, 7.8 Hz, 1H); 6.95–5.88 (m, 2H). ^13^C NMR (100 MHz, DMSO-*d*_6_): δ (ppm) 166.5; 156.7; 130.9; 127.9; 126.2; 121.8; 121.6; 119.6; 119.5; 116.2; 116.2.

#### 2.2.3 [Ni(HL)_2_] (1)

Complex **1** was synthesized by dissolving 193 mg (0.716 mmol) of H_2_L ligand in 10 mL of a mixture (1:1) of ethanol and tetrahydrofuran in a 25 mL round-bottom flask under magnetic stirring and heating to 60 °C. Then, 170 mg (0.716 mmol) of NiCl_2_·6H_2_O was added, resulting in a dark red solution. The reaction mixture was maintained under reflux and magnetic stirring for 4 h. Subsequently, the solution was filtered and then left to allow slow evaporation of the solvents. After a few days, green crystals suitable for single-crystal X-ray diffraction analysis were obtained. Molecular formula C_28_H_20_N_6_NiO_2_S_2_ (595.33 g mol^–1^). Yield: 75%. IR (KBr pellet, cm^–1^): 3203 ν(N–H); 3056 ν(C–H)_ar_; 1597 ν(C=N); 1539 ν(C=N)_ring_; 1267 ν(C–O); 434 ν(Ni–O); 515 ν(Ni–N). UV–vis (DMF; λ_max_ /nm): 295; 358; 423.

#### 2.2.4 [Ni_2_(L)_2_Py_2_(EtOH)]·DMF·0.5H_2_O (**2**)

Complex **2** was synthesized by dissolving 193.0 mg (0.716 mmol) of the H_2_L ligand in 10 mL of a mixture (1:1) of ethanol and chloroform, to which 25 μL of DMF was added. The solution was magnetically stirred and heated at 64 °C in a 25 mL round-bottom flask. Subsequently, 159 μL (1.146 mmol) of Et_3_N was added to the mixture, resulting in a yellowish-brown solution. This was followed by the addition of 340 mg (1.43 mmol) of NiCl_2_·6H_2_O, producing a dark red solution. The reaction mixture was then refluxed for 4 h. After completion, the solution was filtered, and 25 μL (0.310 mmol) of pyridine was added. After a few days of slow solvent evaporation, green crystals suitable for single-crystal X-ray diffraction analysis were obtained. Molecular formula C_43_H_42_N_9_Ni_2_O_4.50_S_2_ (938.39 g mol^–1^). Yield: 69%. IR (KBr pellet, cm^–1^): 3297 ν(O–H); 3062 ν(C–H)_ar_; 2926 ν(C–H)CH_3_; 1664 ν(C=O)_DMF_; 1600 ν(C=N); 1535 ν(C=N)_ring_; 1267 ν(C–O); 488 ν(Ni–O); 504 ν(Ni–N). UV–vis (DMF; λ_max_ /nm): 314; 415.

#### 2.2.5 [Cu_3_(L^#^)_2_(DMF)_2_] (**3**)

Complex **3** was synthesized by dissolving 193 mg (0.716 mmol) of the H_2_L ligand in 10 mL of methanol. The solution was stirred magnetically and heated at 60 °C in a 25 mL round-bottom flask. Subsequently, 100 μL (0.716 mmol) of Et_3_N was added to the mixture, resulting in a slight color change from light brown to yellowish-brown. Then, 3 mL methanolic solution of 173.0 mg (0.716 mmol) of Cu(NO_3_)_2_·3H_2_O was slowly added, producing a dark green solution. The reaction was refluxed for 2 h, yielding a dark greenish-black solid. The obtained solid was recrystallized using DMF. After a few days of slow solvent evaporation, green crystals suitable for single-crystal X-ray diffraction analysis were formed. Molecular formula C_34_H_30_Cu_3_N_8_O_6_S_2_ (901.40 g mol^–1^). Yield: 72%. IR (KBr pellet, cm^–1^): 3064 ν(C–H)_ar_; 2904 ν(C–H)CH_3_; 1613 ν(C=O)_DMF_; 1599 ν(C=N); 1554 ν(C=N)_ring_; 1267 ν(C–O); 910 ν(N–N); 536 ν(Cu–O); 481 ν(Cu–N). UV–vis (DMF; λ_max_ /nm): 357; 410; 428.

### 2.3 DNA-Binding Experiments

Calf thymus-DNA (CT-DNA) from Sigma-Aldrich type I, no. D-1501, was used for DNA studies.

#### 2.3.1 Electronic Absorption Titration

Absorption spectral titrations were carried out in a Tris–HCl buffer with a pH of 7.4 at room temperature. The concentration of CT-DNA was determined from the absorption intensity at 260 nm with a value of 6600 M^–1^ cm^–1^.^24^

The solutions of the complexes studied were prepared in a Tris–HCl buffer containing 5% DMSO. In titration experiments, different concentrations of CT-DNA were used (0.0–73.9 μM) for **1**, (0.0–112.0 μM) for **2**, and (0.0–68.1 μM) for **3**, while the concentration of the complexes was kept constant (71.5, 19.1 and 24.7 μM for complexes **1**, **2** and **3**, respectively). It was impossible to perform the DNA spectroscopic titration for the free ligand, as it was not soluble in the media used. To eliminate the absorbance of CT-DNA an equal amount of the same was added to both the compound and the reference solution. The intrinsic binding constant, *K*_b_, for complexes, was determined from the spectral titration data using the following eq 1(25)
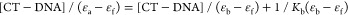
1where [CT-DNA] is the concentration of CT-DNA in base pairs, ε_a_ is the ratio of the absorbance/[complex], ε_f_ is the extinction coefficient of the free complex, and ε_b_ is the extinction coefficient of the complex in the fully bound form. The ratio of the slope to the intercept in the plot of [CT-DNA]/(ε_a_–ε_f_) vs [CT-DNA] gives the value of *K*_b_, which is the calculated absorption band (λ_max_) at around 354–399 nm.

#### 2.3.2 Viscosity Titration Measurements

Viscosity experiments were conducted on an Ostwald viscometer, immersed in a water bath maintained at a constant temperature of 25 °C.^26^ The concentration of CT-DNA in the Tris–HCl buffer is kept constant in all samples, while the concentrations of the compounds were gradually increased, resulting in different molar ratios [complex]/[DNA] from 0 to 0.12. The percentage of DMSO in the assay is kept constant across all samples (5%). The flow time was measured five times for each sample with a digital stopwatch, and the mean flow time was calculated.

Data were analyzed as (η/η_0_)^1/3^ versus the ratio of the concentration of the compound to CT-DNA, where η is the viscosity of CT-DNA in the presence of the compound and η_0_ is the viscosity of CT-DNA alone. Viscosity values were calculated from the observed flow time of CT-DNA-containing solutions corrected from the flow time of buffer (with DMSO) without the complex (*t*-tDNA)/tDNA.^27^

### 2.4 BSA-Binding Experiment

Bovine serum albumin (BSA) from Sigma-Aldrich, no. A-2153, was used for BSA studies.

#### 2.4.1 Fluorescence Spectroscopy

Protein interaction was examined by titration in fluorescence assays. The BSA solution (1.0 μM) was prepared by dissolving the protein in Tris–HCl (pH 7.4) buffer, while the complexes were dissolved in sterile DMSO. In fluorescence measurements, the concentration of BSA (1900 μL) in the Tris–HCl buffer was kept constant in all samples, while the concentration of the complexes (100 μL) was increased to 1.0–6.0 μM. The decrease in the emission intensity of the tryptophan residues of BSA was recorded with excitation at 270 nm and corresponding emission at 345 nm.

The experiments were performed in triplicate and analyzed using the classic Stern–Volmer equation (eq 2)^28,29^
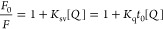
2where *F*_0_ and *F* are the fluorescence intensities in the absence and presence of quencher, respectively, [*Q*] is the quencher concentration, and *K*_sv_ is the Stern–Volmer quenching constant, which can be written as *K*_q_ = *K*_sv_/*t*_0_, where *K*_q_ is the bimolecular quenching rate constant and *t*_0_ is the average lifetime of the fluorophore in the absence of quencher (10^–9^ s). Therefore, eq 2 was applied to determine *K*_sv_ by linear regression of a plot of *F*_0_/*F* versus [*Q*]. The binding constant (*K*_b_) and the number of complexes bound to HSA (*n*) were determined by plotting the double–logarithmic graph of the fluorescence data using eq 3 as follows^28,29^
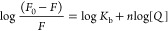
3

### 2.5 Water/1-Octanol Distribution Coefficient (log P)

Water–octanol partition coefficients were determined using the stir flask method.^30^ A total of 1 mg of each complex was solubilized in 100 μL of DMSO (dimethyl sulfoxide). The DMSO solution containing the complex was diluted in a mixture of equal volumes of water (750 μL) and 1-octanol (750 μL). This resulted in the formation of a two-phase system, with the water and 1-octanol layers. The solutions are continuously shaken for 24 h at 1000 rpm and 37 °C. Then the samples were centrifuged for 5 min at 300 rpm and the organic and aqueous phases were separated. The concentration of the ligand and complexes in each phase was measured spectrophotometrically to determine log *P* values using the expression Log *P* = [complex in 1-octanol]/[complex in water].^29^ The experiments were carried out in triplicate.

### 2.6 Antimicrobial Activity

All the synthesized metal complexes (**1**-**3**), along with the ligand (**H**_**2**_**L**), were evaluated for their in vitro antibacterial activity following the guidelines of the Clinical Laboratory Standards Institute (CLSI).^31,32^ The clinical bacterial strains used in the study were obtained from the American Type Culture Collection (ATCC, Rockville, MD, USA), and included *Staphylococcus aureus* (ATCC 25923) and *Bacillus cereus* (ATCC 11778) (Gram-positive), as well as *Salmonella typhimurium* (ATCC 14028) and *Klebsiella pneumoniae* (ATCC 13833) (Gram-negative). In addition, the antifungal activity was assessed against *Candida albicans* (ATCC 90028) and *Candida tropicalis* (ATCC 750).

Bacterial strains were cultured on Müller Hinton agar at 37 °C for 24 h and inoculated with a 10% (v/v) suspension containing 1 × 10^8^ CFU mL^–1^. Yeast strains were cultured on Sabouraud Dextrose agar at 35 °C for 48 h and inoculated with 100 μL of a suspension containing 2.5 × 10^3^ CFU mL^–1^.

Samples underwent serial dilutions in 96-well microplates, using Müller Hinton broth for bacteria and RPMI-1640 broth for yeasts, with final concentrations ranging from 500 to 0.9 μg mL^–1^. For visual determination of the minimum inhibitory concentration (MIC) in bacteria, 50 μL of a 0.1% triphenyl tetrazolium chloride (TTC) solution was added to each well. The MIC was defined as the lowest concentration of the compounds that showed no visible microbial growth.

The minimum bactericidal concentration (MBC) and minimum fungicidal concentration (MFC) were determined by plating aliquots from each well of the microplate onto Müller Hinton agar for bacteria and Sabouraud agar for yeasts. MBC and MFC were defined as the lowest concentrations at which no visible colony growth was observed.

## 3 Results and Discussion

### 3.1 Characterization of the Compounds

#### 3.1.1 NMR Spectra

The ^1^H NMR spectrum of 2-HBT (Figure S1) exhibits signals that correspond to the expected number of hydrogens in the structure, with the integrals matching the hydrogen proportions. The multiplicities observed in the aromatic region are consistent with the presence of a disubstituted ring.^23,33,34^ Similarly, the ^13^C NMR spectrum (Figure S2) displays signals within the characteristic region for aromatic carbons, which aligns with literature values.^23,33^

For H_2_L (Figure S3), the ^1^H NMR spectrum shows signals corresponding to NH, OH, and N=CH protons and aromatic protons. The presence of the imine hydrogen signal confirms the successful condensation reaction between 2-HBT and salicylaldehyde, leading to the formation of the H_2_L ligand.^15^ The ^13^C NMR spectrum of H_2_L (Figure S4) shows signals in the characteristic aromatic carbon region, though some peaks overlap, resulting in fewer distinct signals than expected. The spectrum remains consistent with the ligand structure and literature values.^11,14^

#### 3.1.2 Infrared Spectral

The infrared spectra of the coordination compounds, ligand, and 2-HBT were recorded for their characterization and initial evidence of coordination compound formation. The spectra can be found in Figures S5 and S6. The synthesis of the 2-HBT was confirmed by its IR spectrum, which displayed characteristic absorption bands, including an N–H stretching band of the secondary amine at 3318 cm^–1^, an N–N stretching band at 985 cm^–1^, and two bands corresponding to the primary amine at 3200 and 3129 cm^–1^, consistent with reported values.^13,35^

For the H_2_L ligand, new bands are observed at 1623 cm^–1^ for C=N stretching of the imine group and at 1573 cm^–1^ for C=N stretching of the benzothiazole ring. Other important bands include 1276 cm^–1^ for C–O stretching, 2971 cm^–1^ for phenolic O–H stretching, and 3213 cm^–1^ for N–H stretching, all consistent with literature data for the H_2_L compound.^15,16^

Table 1 summarizes the main vibrational bands and tentative assignments according to the predominant contributions of vibrational modes for the H_2_L ligand and metal complexes **1**, **2**, and **3**. Comparison of these spectra confirms ligand coordination to the metal ions through shifts and the appearance of new bands. The C=N stretching band of the imine in the free ligand (1623 cm^–1^) shifts to lower frequencies (1600–1597 cm^–1^) in the complexes, indicating nitrogen coordination. The presence of the N–H band in complex **1** suggests no ligand deprotonation, while its absence in complexes **2** and **3** confirms deprotonation, consistent with the tautomeric form in complex **3**, as further supported by the bond distances in the SCXRD data. Shifts to lower frequencies in N–N and C–O bands suggest chelation by nitrogen and oxygen atoms.

**Table 1:** Main Peaks Obtained in IR Spectroscopy Compounds **1**, **2** and **3** (cm^–1^)

	H_2_L	1	2	3
ν (N–H)	3213	3203		
ν (O–H)phenolic	2971			
ν (O–H)			3297	
ν (C=O)DMF			1664	1613
ν (C=N)	1623	1597	1600	1599
ν (C= N)ring	1573	1539	1535	1554
ν (N–N)	945	896	907	910
ν (C–O)	1276	1267	1267	1267
ν (M–O)		434	488	536
ν (M–N)		515	504	481

In complexes **2** and **3**, the C=O stretching bands of DMF are observed at 1664 and 1613 cm^–1^, respectively. In complex **2**, DMF acts as a solvate, while in complex **3**, it coordinates directly to the Cu(II) center, causing a shift to lower wavenumbers. Additionally, in complex **2**, a band at 3297 cm^–1^ corresponds to O–H stretching of coordinated ethanol and solvated water, likely overlapping. Lower-frequency bands in the complexes are attributed to interactions between electron-donating atoms and the metal centers, consistent with previous studies.^16,36^

#### 3.1.3 UV–Vis Spectra

The UV–vis characterization of 2-HBT (Figure S7) shows a maximum absorption band in the ultraviolet region at 300 nm, possibly attributed to π → π* electronic transitions in the aromatic ring. In the spectrum of the H_2_L ligand, a similar π → π* band is observed, along with maximum absorption at 358 nm, which may be attributed to *n* → π* electronic transitions, consistent with literature expectations.^11^

The UV–vis absorption spectra of the complexes are shown in Figure S8. These results align with the IR data, confirming complexation, as indicated by the appearance of a characteristic ligand-to-metal charge transfer (LMCT) band.^37^ In complex **1**, this band is subtle, appearing at 423 nm. In complex **2**, the band shows a maximum at 415 nm, and in complex **3**, two potential maxima are observed at 410 and 428 nm. Complexation might also be inferred from the persistence of a band associated with *n* → π* transitions in the aromatic rings, present in both the ligand and complex **1** at 358 nm, as well as from a hypochromic effect, evidenced by a decrease in the peak intensity at 357 nm in the complex **3**.

#### 3.1.4 Single-Crystal X-ray Diffraction (SCXRD)

The complexes **1**–**3** were characterized by SCXRD. Selected bond lengths and angles, and the diffraction intensity collection and refinement data of complexes **1**–**3**, are represented in the Supporting Information.

**Figure 1:**
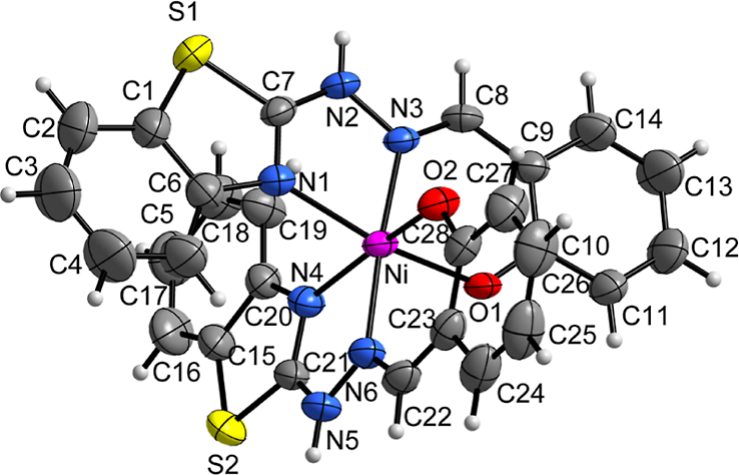
Structural projection of the complex **1**. Anisotropic displacement parameters with 50% occupancy probability.

In the complex [Ni(HL)_2_] (**1**), the nickel center is hexacoordinated by two tridentate ligands in a meridional fashion, involving benzothiazole-*N* (N1 and N4), azomethine-*N* (N3 and N6), and phenolic-O (O1 and O2) donor atoms. A structural representation of the complex, along with the atom numbering scheme, is depicted in Figure 1. The NNO donor sites of the tridentate ligand coordinate the Ni(II) center, forming a five-membered N1–Ni–N3 chelate ring and a six-membered N3–Ni–O1 chelate ring, with bite angles of 79.45(9) and 87.15(8)°, respectively. These angles reflect a distortion from an ideal octahedral geometry. The Ni–O and Ni–N bond lengths range between 2.0 and 2.1 Å, consistent with those reported for similar nickel complexes.^38^ Selected bond lengths and angles for complex **1** are listed in Table S2.

**Figure 2:**
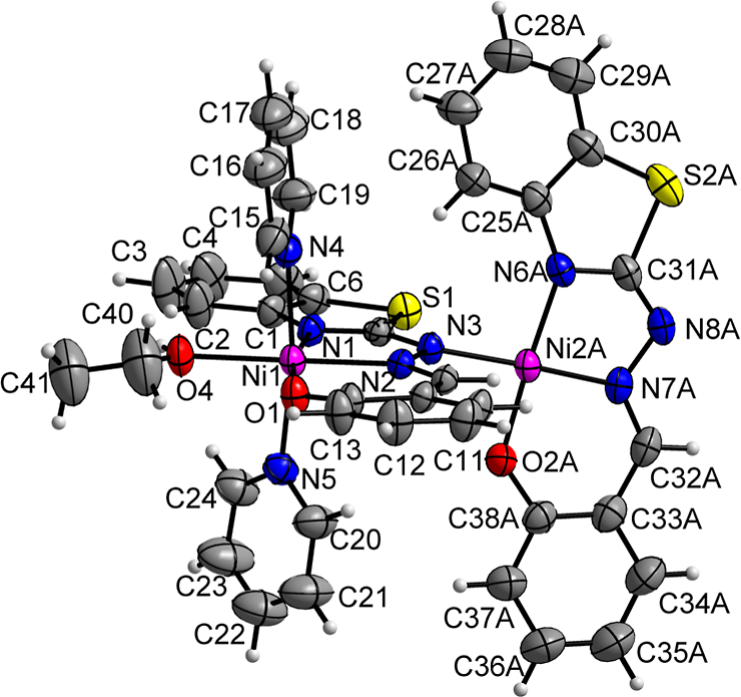
Structural projection of the complex **2**. Anisotropic displacement parameters with 30% occupancy probability. The solvent molecules were omitted for clarity.

The molecular structure of complex **2**, [Ni_2_(L)_2_Py_2_(EtOH)]·DMF·0.5H_2_O, is shown in Figure 2 and reveals a binuclear arrangement. In this structure, the Ni1 atom is coordinated by a tridentate Schiff base ligand (L^2–^), which binds through the benzothiazole nitrogen (N1), azomethine nitrogen (N2), and phenolic oxygen (O1). Additionally, two pyridine molecules coordinate via their nitrogen atoms (N4 and N5), and an ethanol molecule coordinates through its oxygen atom (O4). This results in a hexacoordinated Ni1 center with a slightly distorted octahedral geometry. The nitrogen atoms from the pyridine rings occupy axial positions, with a bond angle N5–Ni–N4 of 171.44(15), and exhibit longer bond distances with Ni compared to the N1 and N2 atoms of the ligand. Selected bond lengths and angles for complex **2** are listed in Table S3.

The second nickel atom (Ni2A) is tetracoordinated, adopting a slightly distorted square planar geometry. Similar to Ni1, one ligand coordinates to Ni2A in a tridentate manner through the N6A, N7A, and O2A atoms. The coordination sphere of Ni2A is completed by the nitrogen atom (N3) of the ligand, which also coordinates with Ni1, thereby bridging the two metal centers. Additionally, SCXRD analysis reveals the presence of two DMF molecules and one water molecule as crystallization solvents for this complex, as shown in Figure S9. The molecular formula notation, specifying 1 DMF molecule and 0.5 water molecule, reflects the half-multiplicity of the solvents.

**Figure 3:**
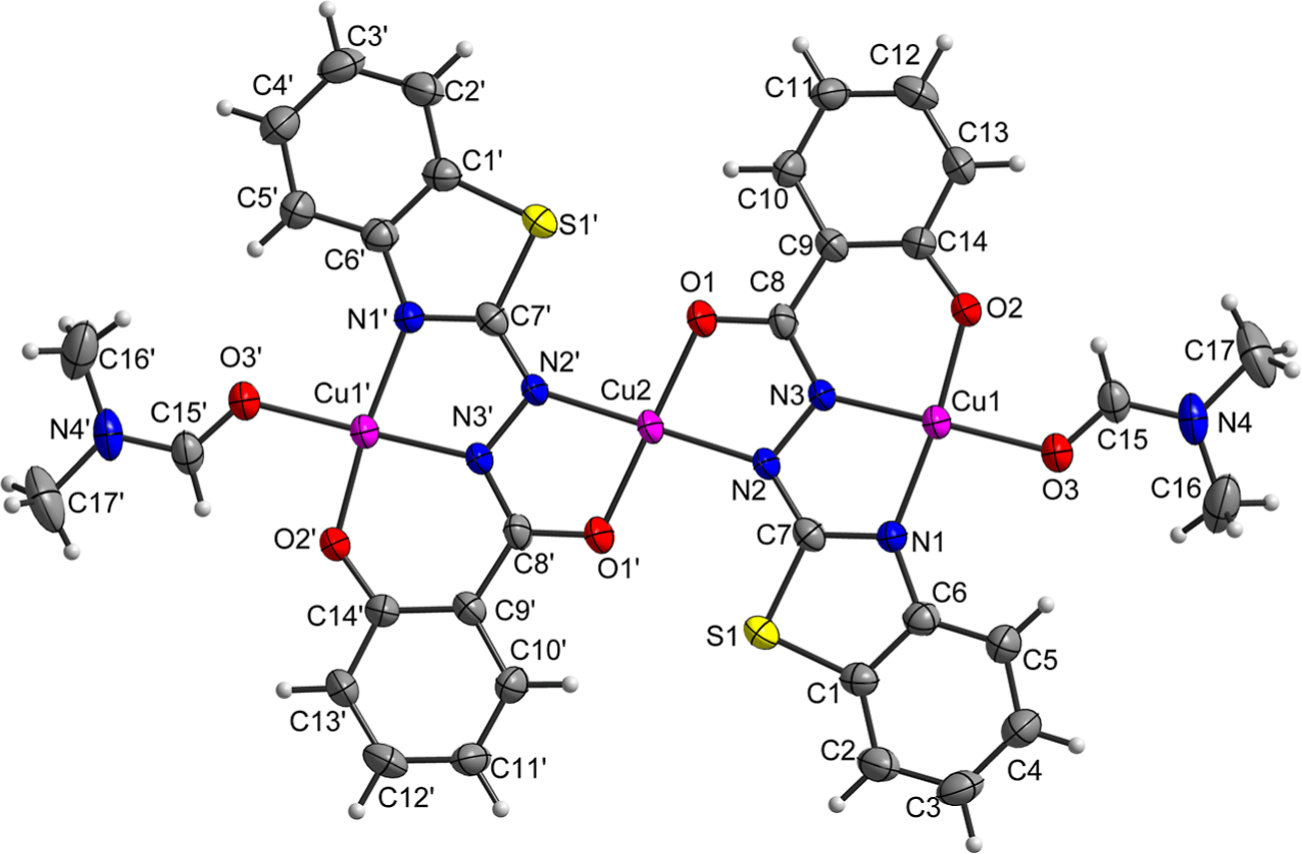
Structural projection of the complex **3**. Anisotropic displacement parameters with 50% occupancy probability. Symmetry code: ’0.5–*x*, −0.5–*y*, 1–*z*.

Complex [Cu_3_(L^#^)_2_(DMF)_2_] (**3**), shown in Figure 3, consists of three copper atoms, each adopting square planar geometry with tetracoordination. The Cu1 atom is coordinated by the oxygen atom (O3) of a DMF molecule and by the ligand through the benzothiazole nitrogen (N1), azomethine nitrogen (N3), and phenolic oxygen (O2), forming five-membered N1–Cu1–N3 and six-membered N3–Cu1–O2 chelate rings with torsion angles of 81.36(12) and 92.39(12)°, respectively, indicating minimal geometric distortion. During synthesis, a structural modification of the ligand was observed, resulting in the formation of an oxygen atom bonded to the imine carbon (C8–O1). This modification enables the formation of a five-membered chelate ring with another metal center (Cu2), involving the imine tautomer nitrogen (N2), with a N2–Cu2–O1 torsion angle of 81.99(11)°. Notably, the Cu2 atom is located at the inversion center of the molecule, with the rest of the structure governed by symmetry (’) 0.5-*x*, −0.5–*y*, 1–*z*.

Table S4 contains the selected bond lengths and angles for the Cu(II) complex. The bond lengths indicate that the ligand adopts the imino tautomeric form. Specifically, the shorter N2–C7 bond compared to the N1–C7 bond suggests that the electron pair is localized outside the thiazole ring, which is consistent with the findings reported by Calinescu et al.^16^

Furthermore, through SCXRD analysis, hydrogen interactions between the ligand molecule and the DMF molecule can be observed, see Table S5. Specifically, there is an interaction between the phenolic oxygen of the ligand and the carbon atom of the DMF molecule (C15–H15^•••^O2) with a bond length of 2.773(5) Å and an angle of 119.75(26)°. Additionally, an interaction occurs between the oxygen atom of the DMF and an aromatic carbon of the ligand (C5–H5^•••^O3), with a bond length of 3.175(5) Å and an angle of 134.803(2)°. According to the classification proposed by Steiner and Jeffery, these can be considered moderate interactions.^39,40^

#### 3.1.5 Thermal Study

**Figure 4:**
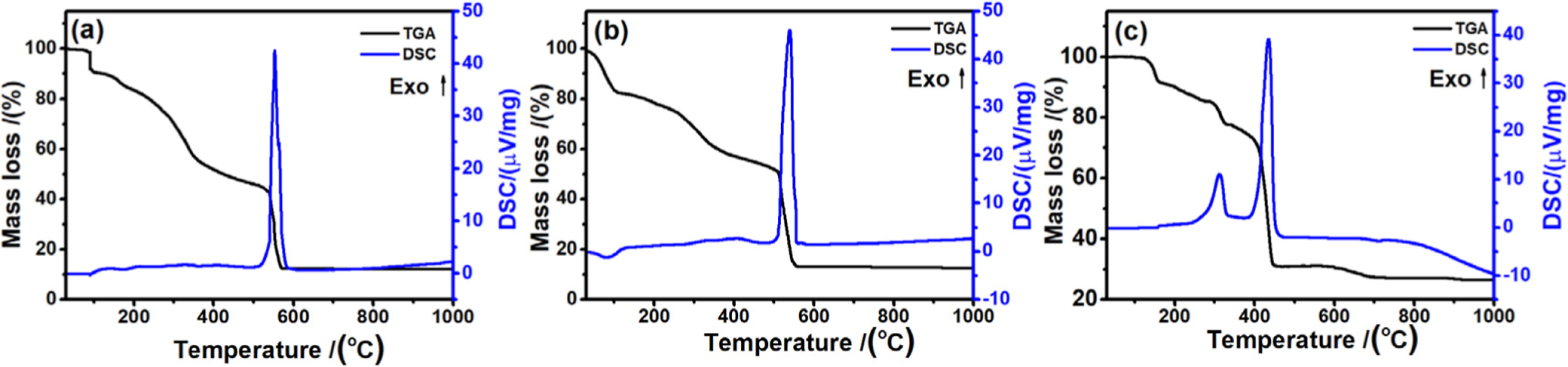
(a–c). TGA-DSC curves for the complexes (a) **1** ([NiL_2_]), (b) **2** ([Ni_2_(L)_2_(Py)_2_(EtOH)]·DMF·0.5H_2_O) and (c) **3** ([Cu_3_(L^#^)_2_(DMF)_2_]) obtained in air atmosphere.

The TGA-DSC curves for compounds are shown in Figure 4a–c. The thermal behavior of each material has different characteristics and, therefore, will be discussed separately for better understanding supported by the SCXRD crystallographic data. However, before starting the discussion it is necessary to take into account the binding energies (kJ mol^–1^) for some important bonds, as these will serve as a parameter to associate the ligand fragmentations with the mass losses observed in the TGA-DSC curves. The main binding energies can be summarized for C=N (615 kJ mol^–1^); C=C (614 kJ mol^–1^); N=N (514 kJ mol^–1^); C–C (348 kJ mol^–1^); C–O (341 kJ mol^–1^); C–N (308 kJ mol^–1^); C–S (260 kJ mol^–1^); N–N (167 kJ mol^–1^). Using these theoretical data for the complex **1** ([NiL_2_], Figure 4a), which has two identical ligands, the first stage of thermal decomposition in TGA begins at approximately 81 °C and ends at 97 °C with a mass loss of 9.0%, which corresponds to the fragmentation of the open chain in the N–N and C–C bonds, releasing fragments of C=N, corresponding to the mentioned percentage. Although the S–C bond has lower energy, sulfur is part of the cyclic chain. The next step in the sequence, occurring between 97 and 174 °C (5.70%), suggests that this mass loss may be associated with the loss of oxygen coordinated to the metal, accompanied by an endothermic event in the DSC at 173 °C. The third (24.5% in TGA) and fourth (16.0% in TGA) steps correspond, respectively, to the decomposition of the sulfur-containing heterocyclic ring and the gradual decomposition of aromatic rings, ultimately leading to the formation of carbonaceous material. The last stage (32.4% TGA) is very characteristic of the combustion/oxidation of the carbonaceous material signaled by a strong exothermic event in DSC with a peak at 552 °C and formation of NiO, as the final residue (12.4% TGA).

For complex **2** ([Ni_2_(L)_2_(Py)_2_(EtOH)]·DMF·0.5H_2_O, Figure 4b), with stoichiometry determined by SCDRX analysis (Figures 2 and S9), the first mass loss of approximately 17%, observed in the TGA curve up to 104 °C and accompanied by an endothermic peak in the DSC at 80 °C, is attributed to the loss of solvated and coordinated ligands. Specifically, this involves one molecule of dimethylformamide (DMF), 0.5 molecule of water, and likely one molecule of pyridine (Py) coordinated to the metal center. In this context, it is plausible to assume that the decomposition of the coordinated ligands begins with pyridine rather than the ethanol molecule. This hypothesis is supported by the fact that Ni–O bonds tend to be shorter and energetically more favorable compared to Ni–N bonds, which are slightly longer and less stable under similar conditions. This is consistent with findings, such as those reported in Chemical Science regarding nickel-mediated N–N bond formation and the stability of oxygen and nitrogen-based ligands.^41^ From 104 °C to approximately 500 °C, the remaining material, [Ni(L)_2_(Py)(EtOH)], decomposes via a more complex pathway. This involves the fragmentation of the open-chain ligand alongside the degradation of pyridine and ethanol, with no detectable thermal events in the DSC. As observed in the TGA, this decomposition occurs gradually, resulting in the formation of a carbonaceous residue that oxidizes or burns starting at 502 °C. This final decomposition is marked by an intense exothermic peak in the DSC at 539 °C, ultimately leading to a stable residue of 13.2%, as nickel oxide (NiO).

For the copper complex **3** ([Cu_3_(L^#^)_2_(DMF)_2_], Figure 4c), no solvated molecules are present. Consequently, its decomposition begins at higher temperatures. According to the molecular structure determined by SCXRD data, two dimethylformamide (DMF) molecules are observed. However, in the TGA curve, the first mass loss (8%) corresponds to the loss of one DMF molecule. Between 161 and 281 °C, the mass loss of the copper complex occurs differently from that of the nickel complexes. In the copper complex, the ligand undergoes structural modifications, including the appearance of an additional hydroxyl group and the presence of the imino-tautomeric form. These changes involve a shift in the positions of the double bonds. Therefore, the decomposition of the complex occurs in a particular way, in which the lowest energy bonds between the N–N and C–C atoms (open chain), can theoretically lead to a mass loss (around 6%) consistent with the N=C–O, also including the loss of oxygen linked to the aromatic ring. After 281 °C, a small plateau of thermal stability is observed up to 289 °C, which is followed by the third stage of mass loss, with a loss of 8% (TGA) signaled by an exothermic event in the DSC at 312 °C. This loss of mass is consistent with the thermal degradation of a DMF molecule coordinated to the metallic center. In the fourth stage of mass loss, between 333 and 387 °C, a slow loss of mass is observed, which is due to decomposition and formation of carbonaceous residue from the aromatic skeleton. The combustion of this material is well signaled in the fifth stage of mass loss with an intense exothermic peak in DSC at 435 °C. This decomposition stage leads to the formation of a stable residue of 31.1%, composed of copper sulfide (CuS), according to stoichiometric calculations. This product is stable up to 588 °C, decomposing completely up to 696 °C, forming 27.2% CuO as final residue.

### 3.2 DNA-Binding Experiments

#### 3.2.1 Electronic Absorption Titration

**Figure 5:**
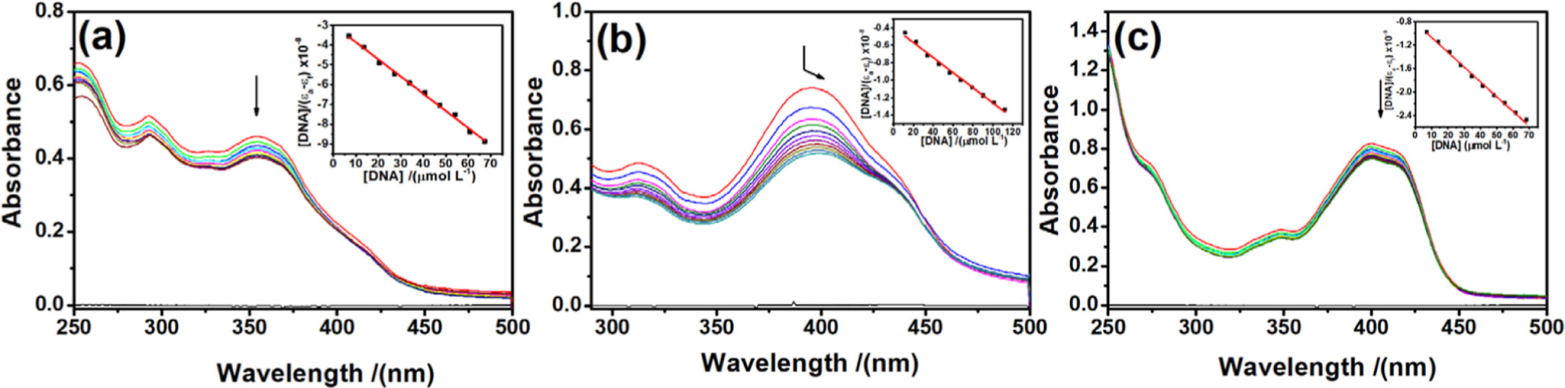
Absorption spectra of complexes (a) **1**, (b) **2** and (c) **3** in the presence of increasing amounts of CT-DNA. The arrow indicates the changes in absorbance upon increasing DNA concentration (insets: plots of [DNA] vs [DNA]/(ε_a_–ε_f_)).

Absorption spectroscopy is one of the most useful techniques to study the binding of any drug to DNA. The changes in the specific bands of a compound, either in intensity (hypochromism/hyperchromism) or displacement (bathochromism/hypsochromism), are used to study the binding of metal complexes to CT-DNA.^42,43^ Absorption spectra of complexes **1**, **2**, and **3** in the presence of increasing amounts of DNA are shown in Figure 5.

The electronic absorption spectra of the complex **1** present a band at 354 nm exhibiting hypochromism of 12.5% upon the addition of DNA, and the band at 394 nm for the **2** complex shows hypochromism of 30.0%, together with a 6.0 nm red shift in wavelength. Moreover, the band at 399 nm for complex **3** exhibits a hypochromism of 9.5%. The spectroscopic changes suggest that the complexes interact with DNA most likely through an intercalation mode, involving stacking interaction between the aromatic chromophores of the complexes and DNA base pairs, due to π–π* stacking interaction. The red shift is evidence of the stabilization of the CT-DNA duplex.^44,45^

The magnitudes of intrinsic binding constants (*K*_b_) were calculated to be 2.90× 10^4^ M^–1^, 2.25 × 10^4^ M^–1^, and 3.03 × 10^4^ M^–1^ for the complexes **1**, **2**, and **3**, respectively. The *K*_b_ values reveal that the copper(II) complex binds more strongly with CT-DNA than the other complexes. This difference can be attributed to the molecular structure of the complexes, as the copper(II) complex has a greater number of coplanar aromatic rings, which appears to enhance the DNA intercalation ability, as seen in other complexes.^43,46,47^

#### 3.2.2 Viscosity Titration Measurements

To further elucidate the binding modes of the complexes with DNA, viscosity measurements were conducted. Viscosity measurements sensitive to length changes are considered the least ambiguous and most critical tests of the binding model in solution.^48^

**Figure 6:**
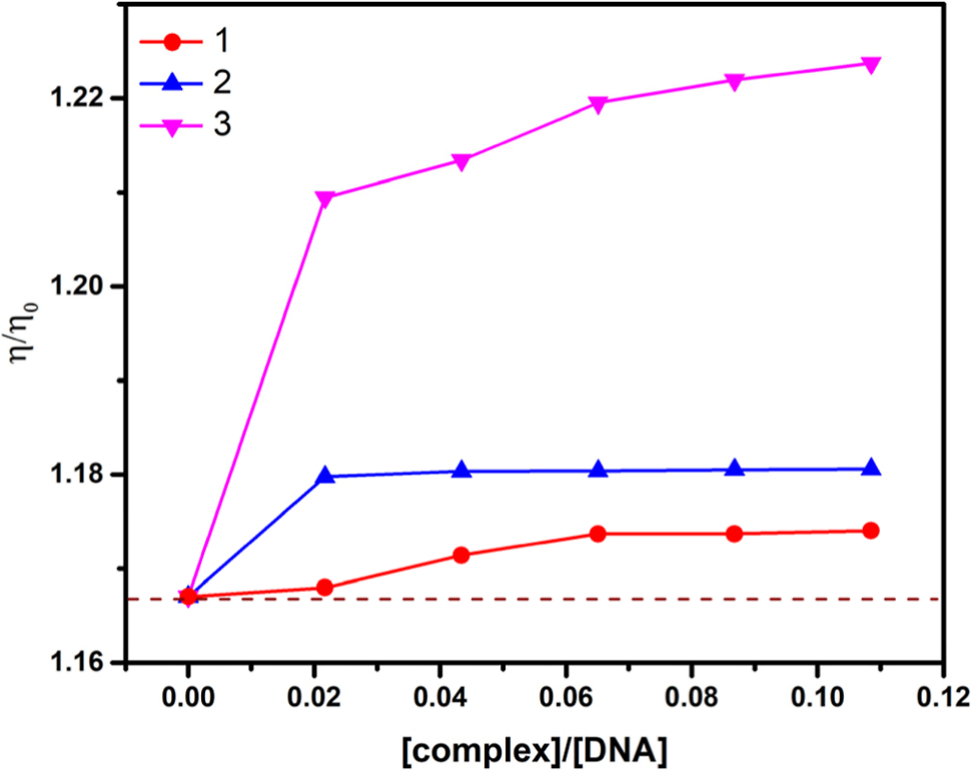
Effect of increasing the amount of complexes on the relative viscosity of CT-DNA at 25.0 ± 0.1 °C. [CT-DNA] = 375 μM, pH = 7.4.

Typically, classical intercalation increases the relative viscosity of DNA because the binding compounds separate the base pairs of the double helix, thus lengthening the DNA helix. Conversely, partial and/or nonclassical intercalation of compounds would reduce the relative viscosity of DNA, as the binding ligand may bend or kink the DNA helix, reducing its effective length. Groove binding or electrostatic interactions can also bend or kink the DNA helix, effectively having no significant impact on DNA viscosity. The values of (η/η_0_)^1/3^ against [complex]/[DNA] are shown in Figure 6 to illustrate the effect of increasing compound concentrations on DNA viscosity.^49,50^

As observed, the increasing [complex]/[DNA] ratios for complexes **1**, **2**, and **3** exhibit a tendency to increase relative viscosity, strongly suggesting intercalation as the primary binding mode of these complexes when interacting with CT-DNA. Notably, the copper(II) complex caused a more pronounced increase than the nickel(II) complexes, indicating stronger binding to DNA through intercalation due to the planarity of its ring system, which aligns with the results observed in the absorption titration curves.^47^

### 3.3 BSA-Binding Experiment

#### 3.3.1 Fluorescence Spectroscopy

**Figure 7:**
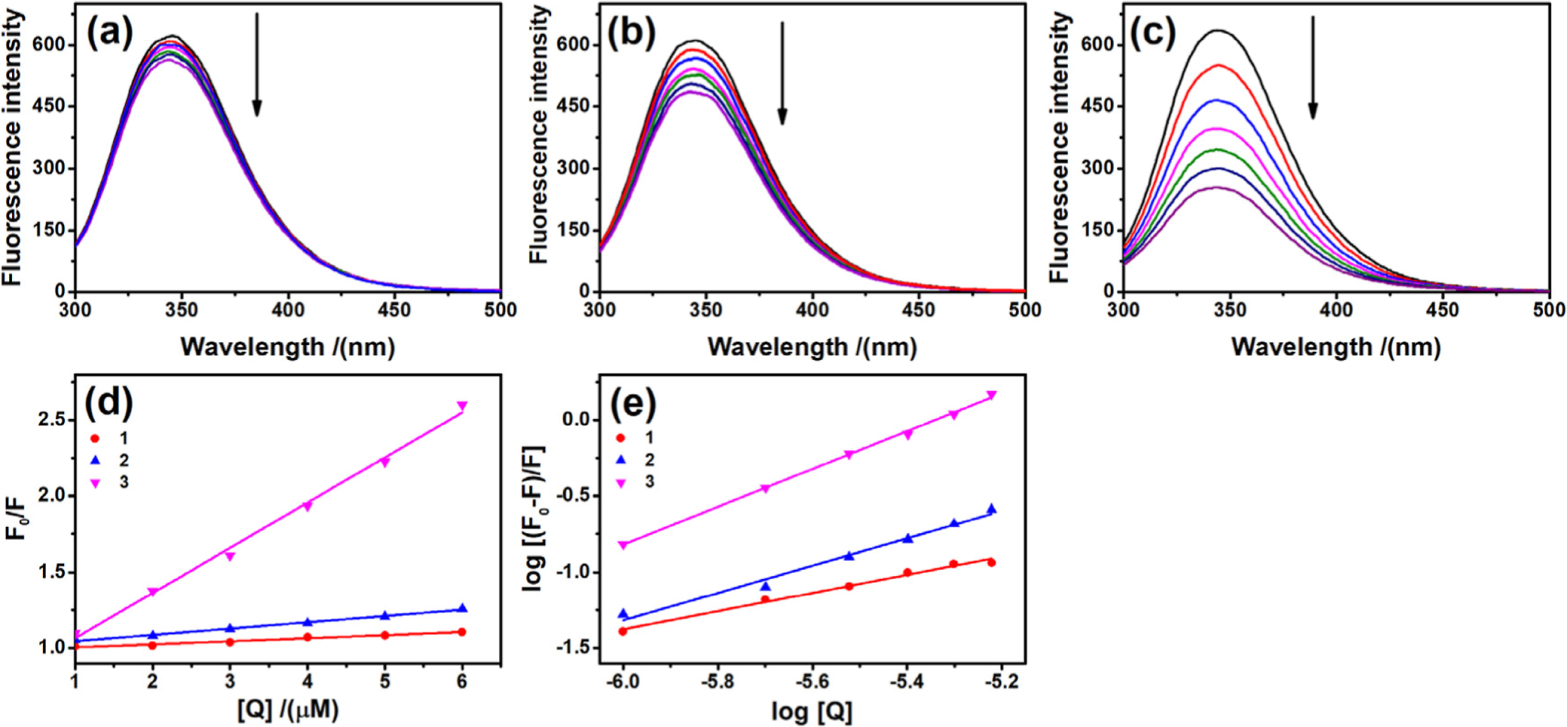
Suppression of BSA fluorescence against different concentrations of the compounds (a) **1**, (b) **2**, (c) **3**. (d) Stern–Volmer plots and (e) Scatchard plots of the fluorescence titration of complexes **1**, **2** and **3** with BSA.

BSA plays a crucial role in drug biodistribution, transport, release, and toxicity. Therefore, we evaluated complex-protein interactions to gain an initial understanding of the pharmacokinetics of the compounds. In this experiment, we observed that the fluorescence intensity of BSA gradually decreased with increasing concentrations of the complexes, indicating that the microenvironment of the BSA Trp-214 residue was affected (Figure 7).^51^

Compounds **1** and **2** showed a *K*_b_ of around 10^3^–10^4^ M^–1^, and, while compound **3** showed a *K*_b_ of around 10^7^ M^–1^. The magnitude of the BSA-binding constant of **1** and **2** and, compared with other compounds reported recently, suggests a moderate interaction with the BSA molecule, while the magnitude of the BSA-binding the compound **3** suggests a strong interaction with the BSA molecule.^52^ These results suggest that they bind to the albumin in a single specific site since the n values are approximately 1 (Table 2). To assess the BSA fluorescence quenching mechanism, we determined the Stern–Volmer constant, suggesting that the BSA fluorescence quenching mechanism was static. Furthermore, the bimolecular quenching constant (*k*_q_) values exceeded 1 × 10^10^ M^–1^ s^–1^, which is above the maximum value for a mechanism to be considered purely dynamic quenching.^52−55^

**Table 2:** BSA–Compound Interaction Constants

compounds	*K*_sv_ (10^4^ M^–1^)	*K*_q_ (10^12^ M^–1^ s^–1^)	*K*_b_ (M^–1^)	*n*
complex **1**	1.72 ± 0.28	1.72	(6.21 ± 0.28) × 10^3^	0.91
complex **2**	3.97 ± 0.21	3.97	(1.02 ± 0.07) × 10^4^	0.90
complex **3**	28.30 ± 2.09	28.30	(1.61 ± 0.08) × 10^7^	1.35

### 3.4 Water/1-Octanol Distribution Coefficient (Log P)

Lipophilicity is typically expressed by the partition coefficient (log P), a molecular parameter that describes the equilibrium of a solute molecule partitioning between water and an immiscible lipid-like organic solvent (1-octanol). The log *P* values determined for the compounds were −0.14 ± 0.02 (**H**_**2**_**L**), −0.21 ± 0.06 (**1**), 0.63 ± 0.13 (**2**), and 0.95 ± 0.11 (**3**). The positive log *P* values for complexes **2** and **3** indicate their relative affinity for the lipid-like organic phase. In contrast, the negative log *P* values for the ligand and the **1** complex indicate their affinity for the aqueous phase or hydrophilicity. Partition coefficients are useful for estimating drug distribution within the organism. It is known that hydrophobic drugs with high partition coefficients are preferentially distributed in hydrophobic compartments, such as the lipid bilayers of cells, while hydrophilic drugs (with low partition coefficients) are preferentially localized in hydrophilic compartments, such as blood serum.^56−58^

### 3.5 Antimicrobial Activity

The antimicrobial activity of the synthesized complexes and the ligand was evaluated against Gram-positive and Gram-negative bacteria, as well as yeasts, as presented in Table 3. The antimicrobial activity of Schiff base metal complexes is influenced by several factors, including the physicochemical structure of the compounds (such as the type of metal and ligand, coordination sites, hydrophilicity, and lipophilicity), the concentration of the tested compound, the type of microorganism, and the testing medium used.^59^ Chelation of the ligand with metal ions is a key factor in enhancing the antimicrobial activity of the complexes, as it increases electron delocalization over the chelate ring, reduces the polarity of the metal ion, and enhances the lipophilic character of the compound, which facilitates its permeation through the cell membrane. This process allows metal complexes to block metal-binding sites in microbial enzymes, resulting in antimicrobial effects.^60−63^

**Table 3:** Minimum Inibitory Concentration (MIC) and Minimum Bactericidal (MBC)/ Fungicidal (MFC) Concentration Values of Compounds (μg mL^–1^)

		ligand (H_2_L)		complex 1		complex 2		complex 3	
antibacterial activity		MIC	MBC	MIC	MBC	MIC	MBC	MIC	MBC
gram positive	*S. aureus*					500		15.6	
	*B. cereus*							31.2	
gram negative	*K. peumoniae*							500	
	*S. typhimurium*							500	
**antifungal activity**		MIC	MFC	MIC	MFC	MIC	MFC	MIC	MFC
*C. albicans*				250	500	125	500	31.2	
*C. tropicalis*				500		500			

In this study, complex **3** stood out, showing the best inhibitory concentrations and being the only compound that exhibited inhibitory activity against all the tested bacteria. Notably, this complex performed even better against Gram-positive bacteria, while complex **2** responded to only one Gram-positive bacterium. This difference can be attributed to the distinct structure of the cell walls in Gram-positive and Gram-negative bacteria. The more complex outer membrane of Gram-negative bacteria may have limited the penetration of the complexes, whereas Gram-positive bacteria, which lack an outer membrane, may be more susceptible to the infiltration of hydrophobic compounds,^64^ as observed for complexes **2** and **3** in Section 3.4.

Remarkably, the copper(II) complex (**3**) demonstrated superior antibacterial activity compared to the nickel(II) complexes (**1** and **2**). This behavior is consistent with previous studies,^63,65,66^ which indicate that copper can bind to amine and carboxyl groups on the surface of Gram-positive bacteria, enhancing its inhibitory potential. Furthermore, copper ions can attack nucleophilic components of the bacterial cell membrane, nucleic acids, or proteins, ultimately leading to bacterial destruction.^67^

In terms of antifungal activity, the ligand showed no significant activity, while the complexes exhibited varying effects against *C. albicans* and *C. tropicalis*. For *C. albicans*, complex **3** displayed the strongest activity, followed by complexes **2** and **1**. Against *C. tropicalis*, both complexes **1** and **2** showed antifungal activity.

## 4 Conclusion

We synthesized three new monocrystalline nickel and copper complexes, which were characterized by infrared spectroscopy, ultraviolet–visible spectroscopy, thermal analysis, and single-crystal X-ray diffraction. This allowed us to determine the structural arrangement, coordination sphere, oxidation number and thermal behavior of all complexes. Based on a precise understanding of the structural design of the complexes, biological studies were conducted with calf thymus DNA (CT-DNA) and bovine serum albumin (BSA). The results provide evidence that interactions with CT-DNA occur in an intercalative manner, classified as moderate based on the binding constant (*K*_b_) obtained from spectroscopic titration and viscosity measurements. Additionally, partition coefficient studies of the compounds, including the ligand, provided information about their potential distribution within the organism, aiming for future pharmaceutical applications. The results indicate that complexes **1** and **3** tend to be distributed in the organic phase, suggesting their ability to cross lipid membranes, while the ligand and complex **2** are more likely to remain in the blood due to their hydrophilic nature. Regarding the interaction with albumin, compounds **1** and **2** showed moderate BSA-binding constants, while compound **3** exhibited much stronger binding. Additionally, the n value close to 1 suggests that all compounds bind to a single specific site on albumin. The fluorescence quenching mechanism of BSA was found to be static, supported by the Stern–Volmer constant and bimolecular quenching constant (*k*_q_) values. The antimicrobial activity of the synthesized complexes and the ligand was tested against bacteria and yeasts. Complex **3** showed the strongest antibacterial effects, especially against Gram-positive bacteria, while the other compounds were less effective. The copper(II) complex outperformed the nickel(II) complexes, likely due to copper’s enhanced ability to penetrate bacterial cells. In antifungal tests, the ligand was inactive, but the complexes showed varying degrees of activity against Candida species.

## Supporting Information Available

^1^H and ^13^C NMR spectra of the precursor and ligand, Infrared and UV–vis spectra of all compounds, and X-ray diffraction data including selected bond lengths and angles for the complexes (PDF)
(CIF)
(CIF)
(CIF)

## Supplementary Material

ao5c00069_si_001.pdf

ao5c00069_si_002.cif

ao5c00069_si_003.cif

ao5c00069_si_004.cif
