# Supplementary material for: Synthesis and Characterization of Novel Hydrazone Complexes: Exploring DNA/BSA Binding and Antimicrobial Potential
Source: ACS Omega. 2025 Feb 13;10(7):7428–40. doi: 10.1021/acsomega.5c00069 (PMC11866212; doi:10.1021/acsomega.5c00069)
Supplement: Supplementary file 1 — ao5c00069_si_001.pdf [file ao5c00069_si_001.pdf]

Supporting Information

Synthesis and Characterization of Novel Hydrazone Complexes: Exploring DNA/BSA Binding and Antimicrobial Potential

Jeniffer Meyer Moreira[1,*], Sara dos Santos Félix Vieira[2], Gabriel de Deus Correia[2], Leandro Nascimento de Almeida[2], Simone Finoto[1], Cândida Alíssia Brandl[3], Aujenus Albert Msumange[1], Fernanda Galvão[4], Kelly Mari Pires de Oliveira[4], Guilherme Caneppele Paveglio[4,†], Monize Martins da Silva[4,‡], Bárbara Tirloni[3], Cláudio Teodoro de Carvalho[1], Daiane Roman[2]

[1]*Quality Control and Thermal Analysis Laboratory, Federal University of Grande Dourados, Dourados, MS 79804-970, Brazil*
[2]*Molecular Synthesis and Modification Laboratory, Federal University of Grande Dourados, Dourados, MS 79804-970, Brazil.*
[3]*Department of Chemistry, Federal University of Santa Maria, Santa Maria, RS 97105-900, Brazil*
[4]*Federal University of Grande Dourados, Dourados, MS 79804-970, Brazil*

[*] Corresponding author's e–mail: jeniffermeyer@hotmail.com
[†] *Hydraulics and Environmental Sanitation Laboratory, State University of Mato Grosso do Sul, Dourados, MS 79804-970, Brazil*
[‡] *State University of Amapá, Macapá, AP 68900-070, Brazil*

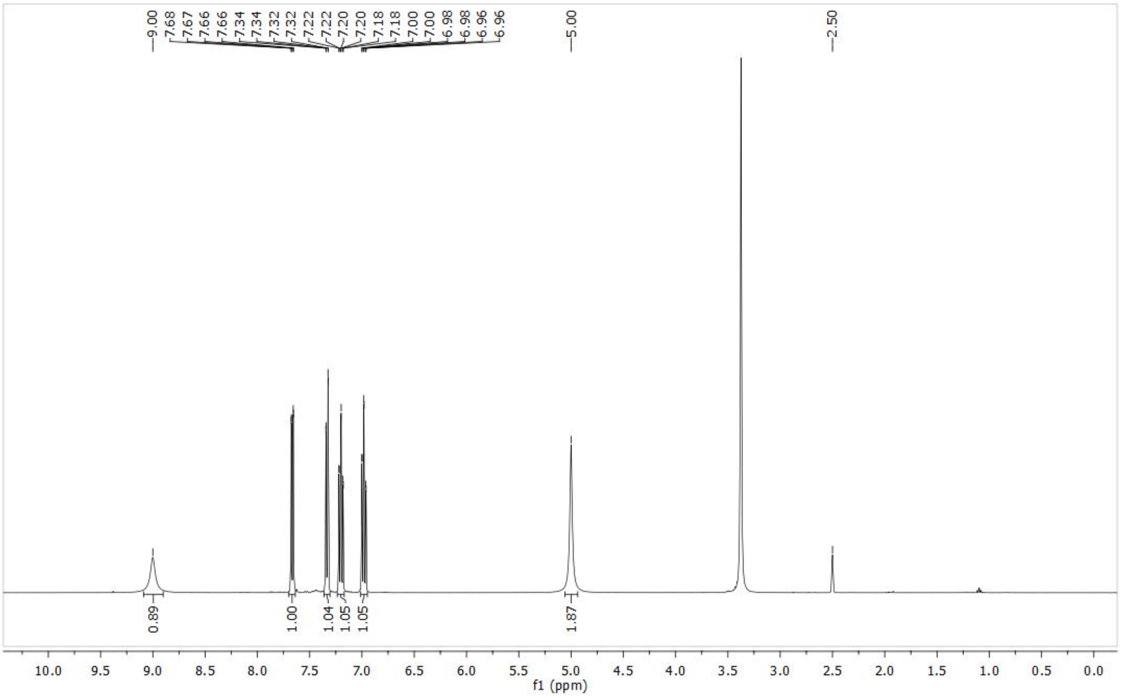

Figure S1. ^{1}H NMR spectrum of 2-HBT.

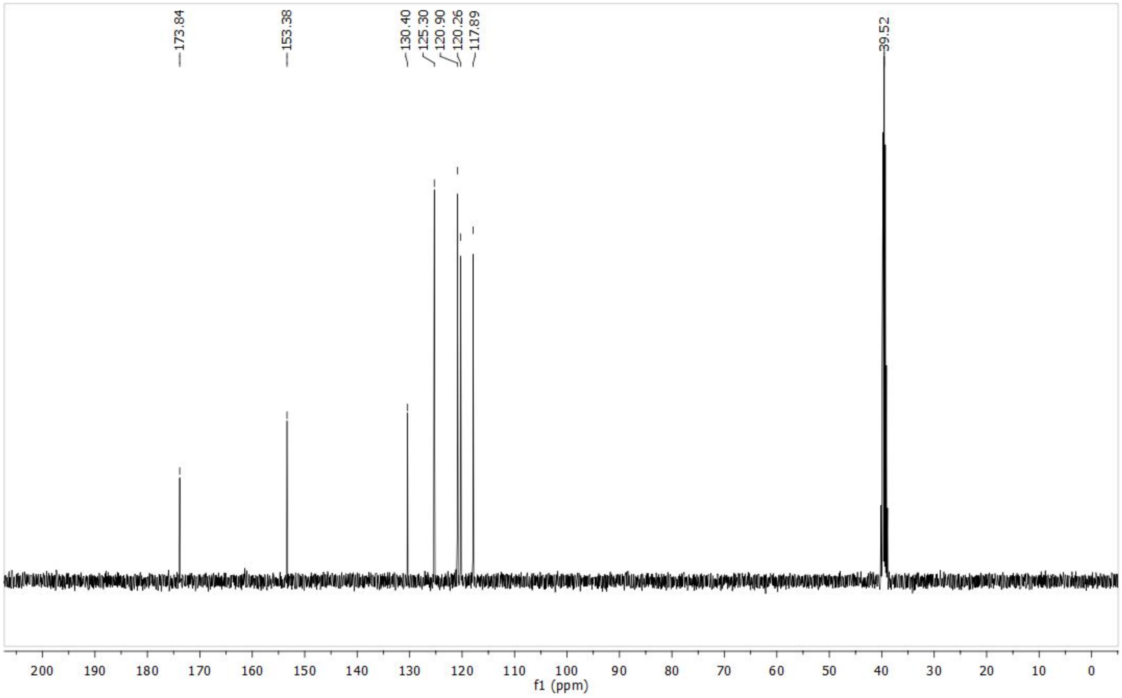

Figure S2. ^{13}C NMR spectrum of 2-HBT.

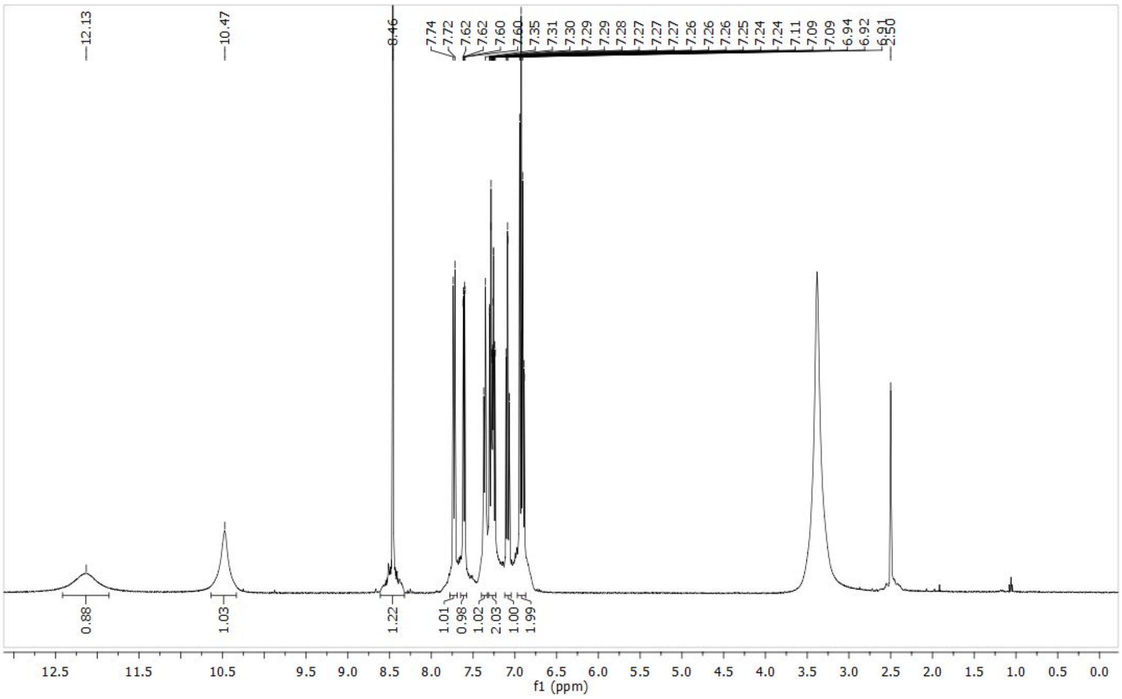

Figure S3. ^{1}H NMR spectrum of H_2L.

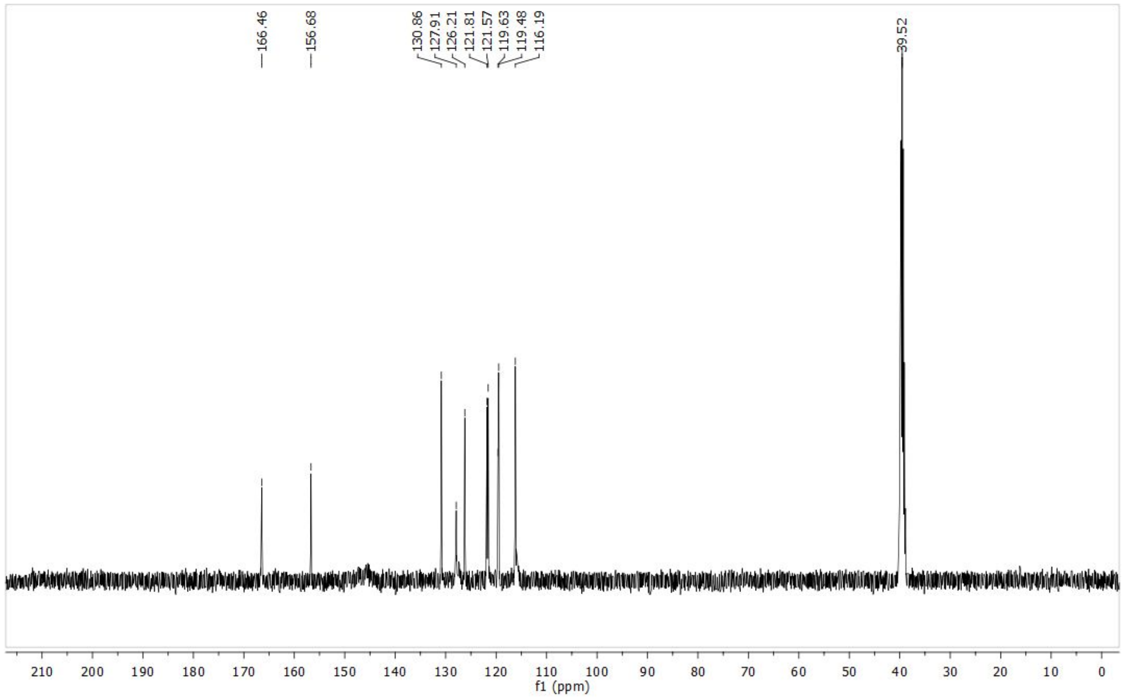

Figure S4. ^{13}C NMR spectrum of H_2L.

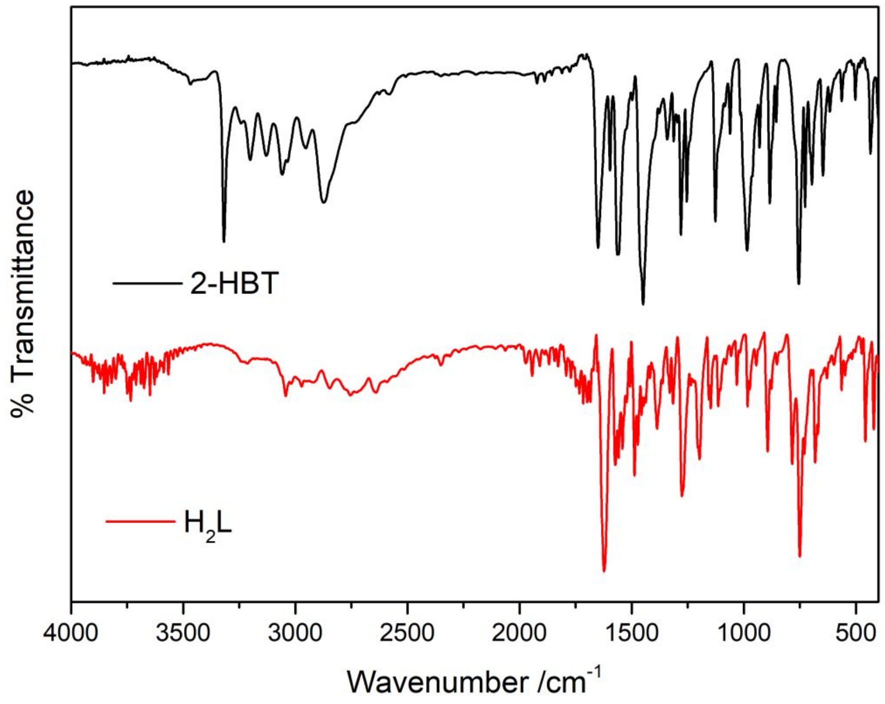

Figure S5. IR spectra of 2-HBT and ligand H_2L.

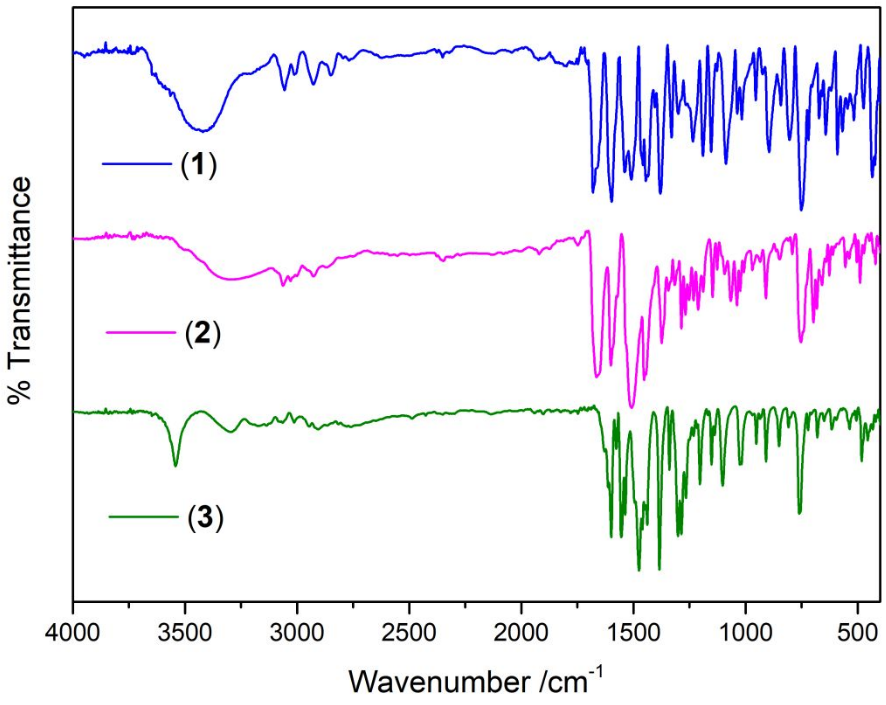

Figure S6. IR spectra of the complexes $[Ni(HL)_2]$ (**1**), $[Ni_2(L)_2(Py)_2(EtOH)]{\cdot}DMF{\cdot}0.5H_2O$ (**2**) and $[Cu_3(L^{\#})_2(DMF)_2]$ (**3**).

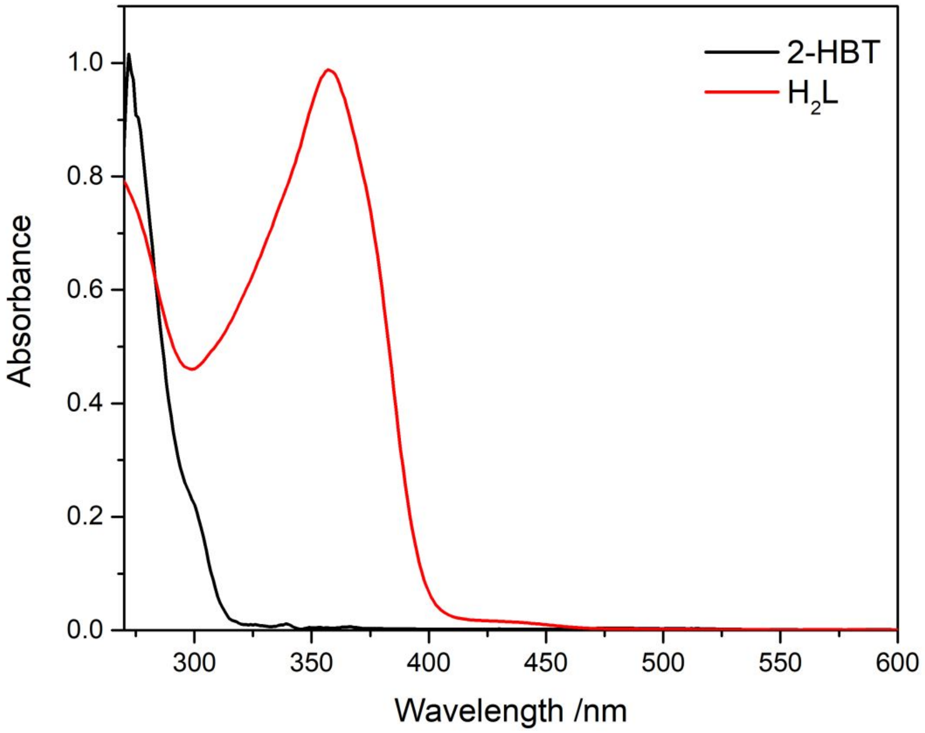

Figure S7. UV-vis spectra of 2-HBT and ligand H_2L.

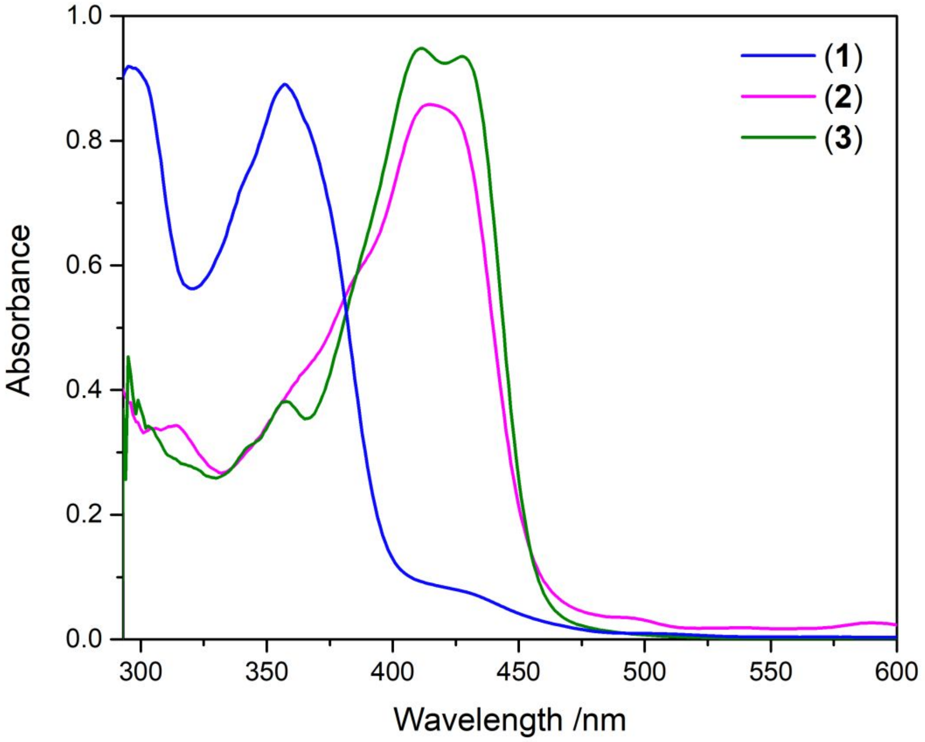

Figure S8. UV-vis spectra of the complexes $[Ni(HL)_2]$ (**1**), $[Ni_2(L)_2(Py)_2(EtOH)]\cdot DMF\cdot 0.5H_2O$ (**2**) and $[Cu_3(L^{\#})_2(DMF)_2]$ (**3**).

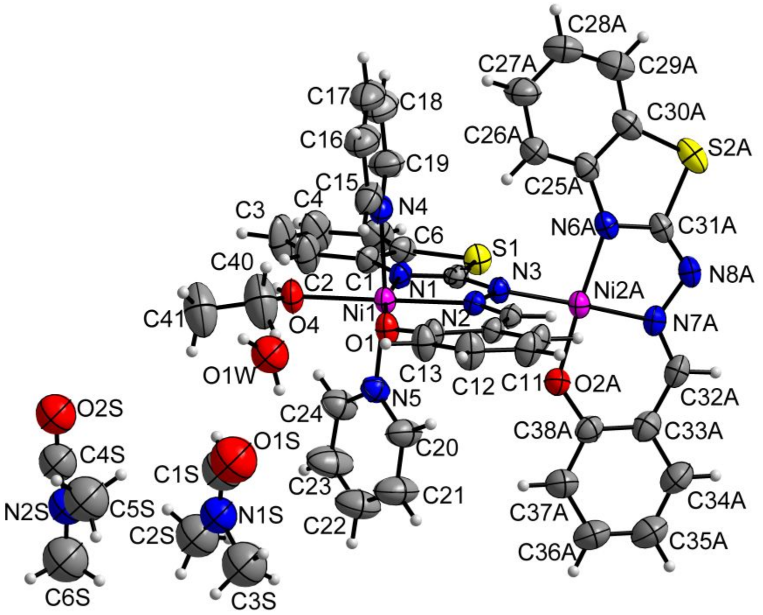

Figure S9. Structural projection of $[Ni_2(L)_2Py_2(EtOH)]\cdot DMF\cdot 0.5\ H_2O$ (**2**). Anisotropic displacement parameters with 30% occupancy probability.

Table S1. Crystal data and structure refinement for complexes **1, 2** and **3**.

Complexes	$[Ni(HL)_2]$ (**1**)	$[Ni_2(L)_2Py_2(EtOH)]\cdot DMF\cdot 0.5 H_2O$ (**2**)	$[Cu_3(L^{\#})_2(DMF)_2]$ (**3**)
Empirical formula	$C_{28}H_{20}N_6NiO_2S_2$	$C_{43}H_{42}N_9Ni_2O_{4.50}S_2$	$C_{34}H_{30}Cu_3N_8O_6S_2$
Formula weight	595.33	938.39	901.40
Temperature (K)	296(2)	296(2)	295(2)
Wavelength (Å)	1.54178	1.54178	0.71073
Crystal system	Tetragonal	Triclinic	Monoclinic
Space group	$P4_1$	$P\bar{1}$	C2/*c*
a (Å)	10.4495(11)	11.781(6)	20.650(3)
b (Å)	10.4495(11)	13.592(4)	6.3965(7)
c (Å)	24.261(5)	16.161(3)	27.599(3)
α (°)	90	102.473(13)	90
β (°)	90	94.100(15)	105.137(4)
γ (°)	90	109.507(12)	90
Volume ($Å^3$)	2649.1(7)	2352.6(15)	3519.1(7)
Z	4	2	4
ρ_{calcd} (mg m^{-3})	1.493	1.325	1.701
μ (mm^{-1})	2.850	2.234	1.974
θ range for data collection (°)	4.607 to 72.112	4.553 to 72.184	2.854 to 30.542
F (000)	1224	974	1828
Crystal size (mm)	0.043 x 0.028 x 0.02[illegible]	0.041 x 0.039 x 0.032	0.124 x 0.065 x 0.050
Index ranges	$-12 \leq h \leq 12$<br>$-11 \leq k \leq 12$<br>$-29 \leq l \leq 29$	$-14 \leq h \leq 14$<br>$-16 \leq k \leq 16$<br>$-19 \leq l \leq 19$	$-28 \leq h \leq 29$<br>$-9 \leq k \leq 8$<br>$-39 \leq l \leq 38$
Reflections collected/independent	17944/5086	41294	17944/5377
Data/ restraints/ parameters	5086/ 1/ 353	9177 / 863 / 665	5377 /0/ 243
Goodness-of-fit on F^2	1.036	1.009	0.996
R_1, wR_2 [$I > 2\sigma(I)$]	0.0255, 0.0640	0.0818, 0.2516	0.0594, 0.0971
R_1, wR_2 (all data)]	0.0271, 0.0653	0.1029, 0.2774	0.1814, 0.1208
$\Delta\rho_{max}$, $\Delta\rho_{min}$ (e $Å^{-3}$)	0.148, –0.241	1.179, –0.851	0.403, –0.384

Table S2. Selected bond lengths (Å) and angles (°) of $[Ni(HL)_2]$ (**1**)

**Bond lengths (Å)**		**Bond angles (°)**	
Ni–O2	2.019(2)	N1–Ni–N4	84.49(9)
Ni–N3	2.031(2)	N4–Ni–N6	78.32(10)
Ni–N6	2.035(2)	N6–Ni–O1	87.81(9)
Ni–O1	2.051(2)	O1–Ni–O2	92.33(9)
Ni–N4	2.107(2)	O2–Ni–N3	90.31(9)
Ni–N1	2.109(2)	N3–Ni–N1	100.75(9)
		O1–Ni–N3	87.15(8)
		N3–Ni–N4	100.75(9)
		N3–Ni–N6	174.81(9)

Table S3. Selected bond lengths (Å) and angles (°) of $[Ni_2(L)_2Py_2(EtOH)]·DMF·0.5\ H_2O$ (**2**).

**Bond lengths (Å)**		**Bond angles (º)**	
Ni1–O1	1.996(3)	O1–Ni1–N2	90.52(12)
Ni1–O4	2.117(3)	N2–Ni1–N1	78.44(12)
Ni1–N1	2.077(4)	O1–Ni1–O4	93.20(15)
Ni1–N2	2.038(3)	N1–Ni1–O4	97.81(14)
Ni1–N4	2.179(4)	O1–Ni1–N5	89.15(14)
Ni1–N5	2.148(4)	N2–Ni1–N5	93.36(14)
Ni2A–O2A	1.833(6)	N1–Ni1–N4	92.94(16)
Ni2A–N7A	1.879(10)	O4–Ni1–N4	85.57(15)
Ni2A–N6A	1.906(6)	N4–Ni1–N5	171.44(15)
Ni2A–N3	1.937(3)	O2A–Ni2A–N7A	94.3(4)
		N7A–Ni2A–N6A	82.8(4)
		O2A–Ni2A–N3	83.8(2)
		N6A–Ni2A–N3	99.2(2)

Table S4. Selected bond lengths (Å) and angles (°) of $[Cu_3(L^{\#})_2(DMF)_2]$ (**3**).

**Bond lengths (Å)**		**Bond angles (°)**	
Cu1–O2	1.865(3)	O2–Cu1–N3	92.39(12)
Cu1–O3	1.986(2)	N3–Cu1–N1	81.36(12)
Cu1–N1	1.963(3)	O2–Cu1–O3	89.65(11)
Cu1–N3	1.910(3)	N1–Cu1–O3	96.93(11)
Cu2–O1	1.927(2)	O1–Cu2–N2	81.99(11)
Cu2–N2	1.943(3)	O1–Cu2–N2’	98.01(11)
N2–C7	1.323(4)		
N1–C7	1.337(4)		

Symmetry code: ’ 0.5-*x*, -0.5-*y*, 1-*z*.

Table S5. Hydrogen bonds (Å and °) for $[Cu_3(L^{\#})_2(DMF)_2]$ (**3**).

D-H···A	d(D-H)	d(H···A)	<(DHA)
C5-H5···O3	0.930	2.450	3.175(5)
C15-H15···O2	0.930	2.191	2.773(5)
